# Relationship of Time-Activity-Adjusted Particle Number Concentration with Blood Pressure

**DOI:** 10.3390/ijerph15092036

**Published:** 2018-09-18

**Authors:** Laura Corlin, Shannon Ball, Mark Woodin, Allison P. Patton, Kevin Lane, John L. Durant, Doug Brugge

**Affiliations:** 1 Department of Civil and Environmental Engineering, Tufts University School of Engineering, 200 College Ave, Medford, MA 02155, USA; shannon.ball94@gmail.com (S.B.); mark.woodin@tufts.edu (M.W.); apatton@healtheffects.org (A.P.P.); john.durant@tufts.edu (J.L.D.); doug.brugge@gmail.com (D.B.); 2 Section of Preventive Medicine and Epidemiology, Boston University School of Medicine, 801 Massachusetts Avenue, Suite 470, Boston, MA 02118, USA; 3 Department of Public Health and Community Medicine, Tufts University School of Medicine, 136 Harrison Avenue, Boston, MA 02111, USA; 4 Health Effects Institute, 75 Federal Street, Suite 1400, Boston, MA 02110, USA; 5 Department of Environmental Health, Boston University School of Public Health, 715 Albany St, Boston, MA 02118, USA; klane@bu.edu; 6 Tufts University Jonathan M. Tisch College of Civic Life, 35 Professors Row, Medford, MA 02155, USA

**Keywords:** particle number concentration, ultrafine particulate matter, time-activity adjustment, blood pressure, hypertension, traffic-related air pollution, directed acyclic graph

## Abstract

Emerging evidence suggests long-term exposure to ultrafine particulate matter (UFP, aerodynamic diameter < 0.1 µm) is associated with adverse cardiovascular outcomes. We investigated whether annual average UFP exposure was associated with measured systolic blood pressure (SBP), diastolic blood pressure (DBP), pulse pressure (PP), and hypertension prevalence among 409 adults participating in the cross-sectional Community Assessment of Freeway Exposure and Health (CAFEH) study. We used measurements of particle number concentration (PNC, a proxy for UFP) obtained from mobile monitoring campaigns in three near-highway and three urban background areas in and near Boston, Massachusetts to develop PNC regression models (20-m spatial and hourly temporal resolution). Individual modeled estimates were adjusted for time spent in different micro-environments (time-activity-adjusted PNC, TAA-PNC). Mean TAA-PNC was 22,000 particles/cm^3^ (sd = 6500). In linear models (logistic for hypertension) adjusted for the minimally sufficient set of covariates indicated by a directed acyclic graph (DAG), we found positive, non-significant associations between natural log-transformed TAA-PNC and SBP (β = 5.23, 95%CI: −0.68, 11.14 mmHg), PP (β = 4.27, 95%CI: −0.79, 9.32 mmHg), and hypertension (OR = 1.81, 95%CI: 0.94, 3.48), but not DBP (β = 0.96, 95%CI: −2.08, 4.00 mmHg). Associations were stronger among non-Hispanic white participants and among diabetics in analyses stratified by race/ethnicity and, separately, by health status.

## 1. Introduction

Ambient particulate matter (PM) exposure is associated with over four million deaths per year and evidence suggests that certain size fractions of PM are associated with increased risk of hypertension, cardiovascular morbidity, and cardiovascular mortality [1,2,3,4,5,6]. Nonetheless, few epidemiologic studies have considered the cardiovascular impacts of long-term exposure to the smallest size fraction of PM, ultrafine particulate matter (UFP, aerodynamic diameter < 0.1 µm). This is despite toxicologic evidence that UFP may be the most toxic PM size fraction and that UFP may exert cardiovascular effects through mechanisms involving oxidative stress and systemic inflammation [7,8,9,10,11,12]. In the few prospective epidemiologic studies that have considered the cardiovascular consequences of long-term exposure to UFP, there has been reasonable agreement that UFP exposure is associated with adverse cardiovascular impacts including increased risk of ischemic heart disease mortality, increased hypertension risk, increased carotid intima-media thickness, and, in some cases, increased concentrations of biomarkers of inflammation [13,14,15,16,17,18]. Nevertheless, most previous studies have only considered UFP modeled with a spatial resolution of between 200 m and 4 km or have considered the cardiovascular impacts of UFP only in relatively homogenous populations. To inform risk assessment and policy development, studies in heterogeneous populations with highly spatially–resolved UFP exposure estimates are needed.

Modeling UFP with insufficiently high spatial resolution can result in substantial exposure misclassification as UFP concentrations rapidly decline within 100 m of sources, such as major roadways [19]. Similarly, exposure assessment methods that rely on residential average concentrations, rather than methods that also account for time individuals spend away from the home, can result in differential exposure misclassification [20]. We are only aware of one prospective study that modeled UFP at fine spatial resolution (≤20 m). This was our longitudinal analysis of the association of long-term exposure to UFP with blood pressure and C-reactive protein within the Boston Puerto Rican Health Study (BPRHS) [16]. Nevertheless, in that study, we could not account for individual time-activity patterns which could have reduced potential exposure misclassification [20]. Additionally, while there were some indications that certain sub-populations were more vulnerable to the effects of UFP, the BPRHS population consisted only of individuals who identified as Puerto Rican and most BPRHS participants were in generally poor overall health. To understand whether the associations we found in our previous study were generalizable to more diverse populations and whether the associations remained after accounting for time spent in different micro-environments, we used data from the Community Assessment of Freeway Exposure and Health (CAFEH) study.

In the cross-sectional CAFEH study, participants of multiple races/ethnicities were recruited from several communities in the greater Boston area. Using measurements of UFP (as particle number concentration (PNC)—a commonly used and reliable indicator of UFP [21,22]) from each community, we developed a finely resolved (hourly temporal and approximately 20 m spatial resolution) model. We adjusted mean hourly residential estimates using individual data on time spent in different micro-environments to assess time-activity-adjusted PNC (TAA-PNC) [20,23]. We previously found that long-term TAA-PNC was associated with biomarkers of systemic inflammation and with chronic outcomes in the CAFEH population [24,25]. Given evidence that these associations with PNC varied by race/ethnicity and that there were racial disparities in overall health status in this population, we considered effect modification by race/ethnicity and by health factors which could affect susceptibility to UFP [24,26,27,28]. Specifically, our objectives were: (1) to determine whether long-term exposure to TAA-PNC was associated with systolic blood pressure (SBP), diastolic blood pressure (DBP), pulse pressure (PP), and prevalent hypertension; and (2) to determine whether race/ethnicity, statin medication use, diabetes status, or hypertension status modified these associations.

## 2. Materials and Methods

The cross-sectional, community-based participatory CAFEH study was designed to investigate the relationship between UFP exposure and cardiovascular health. Detailed methods have been published elsewhere [29]. Briefly, all participants were at least 40 years of age and were able to complete a survey in one of six languages. Participants were recruited from four neighborhoods in the Boston metropolitan area (Somerville, Dorchester/South Boston, Chinatown, and Malden). To maximize exposure contrast, individuals residing <100 m, 100–500 m, and >1000 m of either Interstate 90 or Interstate 93 were recruited as part of a stratified random sample. To increase the sample size, individuals residing in elderly housing developments in Somerville and Dorchester/South Boston and individuals residing in the same buildings and floors as participants in the stratified random sample in Chinatown were recruited as part of a convenience sample (18% of participants were part of the convenience sample). Of the 704 participants who completed in-home surveys, 455 participants also attended a clinic visit where their blood pressure was measured. Of these participants, 409 were included in the present analysis since they had complete information on UFP exposure, self-reported race/ethnicity, and blood pressure outcomes. A secondary analysis was also conducted on the 205 participants from Somerville and Dorchester/South Boston who attended a second clinic visit approximately five months after their initial clinic visit (mean time between visits = 138 days, sd = 53 days, min = 35 days, max = 364 days). The Tufts University Health Sciences Institutional Review Board approved the study (protocol # 8468; originally approved in 2008; most recent approval June 13 2017). All participants gave written informed consent.

### 2.1. Demographics and Health Data

During the in-home visit, participants self-reported age, sex, education (less than high school, high school, or more than high school), race/ethnicity (non-Hispanic white, Asian, or other), country of birth, smoker status (never, former, or current), doctor diagnoses of several health conditions (e.g., hypertension, diabetes), air conditioner use, and time spent in five micro-environments (inside home, outside home, school/work, commuting, and other) on a recent workday/weekday and a recent weekend/non-work day (depending on employment status). Participants also reported frequency of fruit and vegetable consumption (less than or at least seven times per week of both fruits and vegetables), fried food consumption (less than or at least once per week), gas stove use in the home (less than or more than half of the days in the month), and annoyance with traffic sound at home (never, sometimes, often, or always). Participants were also asked to show all of their medications to the field staff member and they were surveyed about physical activity (represented here as the natural log number of minutes per week participants engaged in light or moderate physical activity for consistency with previously published work) [26,30].

At each clinic visit, participants’ height, weight, and seated blood pressure were measured. Body mass index (BMI) was calculated as weight (kg)/[height (m)]^2^. Blood pressure was measured with an automatic blood pressure machine (Model HEM711ACN2; Omron Healthcare, Kyoto, Japan). For SBP and DBP, measurements taken from the left and right arms were averaged. PP was calculated as the difference between SBP and DBP. Participants were classified as hypertensive if they had a measured SBP above 140, a measured DBP above 90, or if they reported taking medications to treat hypertension.

### 2.2. Exposure Assessment

Methods for estimating annual average PNC adjusted for individual time-activity data on time spent in different micro-environments (TAA-PNC) have been published previously [20,23,31,32,33]. Briefly, air pollution monitoring was conducted with the Tufts Air Pollution Monitoring Laboratory (TAPL-1) between September 2009 and July 2012 along a fixed route in each of Somerville, Dorchester/South Boston, Chinatown, and Malden. TAPL-1 is a retrofitted gasoline-powered Class-C recreational vehicle equipped with rapid-response instruments, including a butanol-based condensation particle counter (TSI, Model 3775; 4-3000 nm) used to measure PNC with one second averaging time. Spatial coordinates were assigned using a Garmin V GPS receiver (manufacturer-specified accuracy: 3–5 m) [31]. These data were used along with spatial and temporal covariates (e.g., distance from residence to nearest highway and major road, wind speed, wind direction, temperature, day of week, highway traffic volume, and highway traffic speed) to develop a model estimating hourly natural log PNC values at participant residences for each hour of the year in which the participant attended the initial CAFEH study visit [23,32]. Hourly residential PNC estimates were then adjusted for infiltration of PNC into residences, air conditioner use, and participant time-activity based on the amount of workday/weekday and non-workday/weekend time participants reported spending in different micro-environments (inside home, outside home, at work, on highway, and other) [20,33]. For participants who attended a second CAFEH clinic visit, micro-environment time-activity data were consistent between the two study visits. Additional details are given in Appendix A.

### 2.3. Statistical Analysis

We examined the distribution of demographic factors and exposure estimates in our study population as a whole and stratified by race/ethnicity, statin medication use, and diabetes status. For continuous variables, we calculated the mean and standard deviation. For categorical variables, we calculated proportions. We used independent sample *t*-tests, one-way ANOVA, and chi-square statistics to examine differences by race/ethnicity, statin medication use, and diabetes status.

#### 2.3.1. Conceptual Model

**Figure 1:**
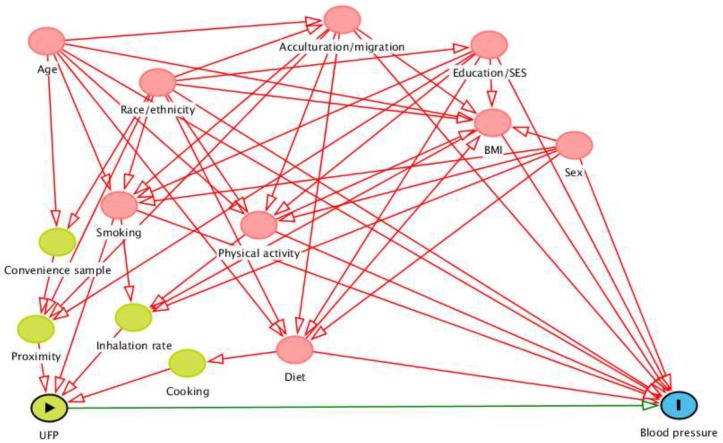
Directed acyclic graph representing the relationships among the exposure (UFP; represented by the green oval with the triangle), outcome (blood pressure; represented by the blue oval with the line), and related factors. Variables represented as pink ovals are ancestors of exposure and outcome while variables represented as green ovals (convenience sample, proximity, inhalation rate, and cooking) are ancestors only of the exposure. Pink lines are biasing paths and the green line between the exposure and outcome is the causal path of interest.

We constructed the directed acyclic graph (DAG) shown in Figure 1 based on an extensive literature review, as detailed in Appendix B. This type of conceptual model makes assumptions explicit and identifies the minimally sufficient set of variables needed to account for confounding of the exposure–outcome relationship [34]. Since UFP exposure was defined largely by participants’ proximity to roadways, we assumed that proximity was accounted for in all models. Based on this assumption and the relationships in Figure 1, there were four possible minimally sufficient sets of covariates: (1) BMI, diet, physical activity, sex, and smoking; (2) BMI, cooking, physical activity, sex, and smoking; (3) cooking, inhalation rate, and smoking; and (4) diet, inhalation rate, and smoking. Since we did not have any direct measure of inhalation rate, we could not use the third or fourth minimally sufficient sets. Additionally, as our main measure for cooking-related exposures was residential use of a gas stove and this seemed like a crude proxy, we prioritized the first minimally sufficient set for all analyses. In modeling and in tests of the DAG, acculturation/migration was represented by a dichotomous variable for nativity (born in the US or not), proximity was represented by distance to the nearest highway, and diet was represented alternatively as fried food consumption and as fruit and vegetable consumption.

#### 2.3.2. Primary Analysis and Sensitivity Analyses

For each of the four outcomes (SBP, DBP, PP, and hypertension), we constructed separate models (linear models for SBP, DBP, and PP; logistic models for hypertension). We first examined the unadjusted associations between natural log-transformed TAA-PNC exposure and each outcome (Model A). Our main model examined the association between natural log-transformed TAA-PNC exposure and each outcome adjusted for covariates in the first minimally sufficient set identified by the DAG (Model B: BMI, sex, smoking, physical activity, and diet as fried food consumption). We tested collinearity using variance inflation factors (all values were <2). We also examined the normality and homoscedasticity of the residuals. For the hypertension model, we examined the Hosmer–Lemeshow goodness-of-fit test (*p* = 0.91).

To assess the sensitivity of this model to the choice of the proxy for diet, we used two other proxies for diet (fruit and vegetable consumption in Model C; and race/ethnicity in Model D). We also assessed whether age was a residual confounder in Model E. Since age is an exogenous variable, its inclusion would not be expected to introduce confounding. To assess the sensitivity of our analysis to the assumption that proximity was accounted for within the exposure value, we tested the association between natural log-transformed TAA-PNC exposure and each outcome adjusting for the covariates in Model B as well as covariates that would serve as three different sets of proxies for proximity (Model F: age, race/ethnicity (assumed to define acculturation/migration status), and educational attainment; Model G: Model F covariates as well as a variable representing inclusion in the convenience sample; and Model H: Model B covariates as well as annoyance with traffic sound).

#### 2.3.3. Effect Modification

We examined the associations between natural log-transformed TAA-PNC exposure and each outcome stratified (separately) by race/ethnicity, statin medication use, diabetes status, and hypertension status (only for blood pressure models). We considered unadjusted models (Model A), models adjusted for the primary covariates (Model B), and models adjusted for the primary covariates as well as age (Model E). While we considered the p value for interactions, these were largely un-interpretable because the analysis was not adequately powered to detect interactions.

#### 2.3.4. Consistency of Associations over Time

To determine if the associations we found were stable over time, we compared the effect estimates for the association of natural log-transformed TAA-PNC exposure with each outcome at clinic visit one and at clinic visit two (*n* = 205). We considered unadjusted models (Model A), models adjusted for the primary covariates (Model B), and models that did not assume that proximity status largely defines exposure status (Model F).

## 3. Results

Of the 409 study participants, the majority were female (59%) and the average age was 62 years (Table 1). Most participants self-identified as non-Hispanic white (*n* = 178) or Asian (*n* = 149). There were 82 participants who self-identified as another race/ethnicity, including 36 black and 26 Hispanic participants. While 87% of the white participants were born in the United States, all of the Asian participants and 59% of the participants of other races/ethnicities were born outside of the United States (Table 1). Most of the Asian participants were born in China (*n* = 126) or Vietnam (*n* = 13). Compared to participants who identified as white or as another race/ethnicity, Asian participants were least likely to have attained at least a high school education (*p* < 0.001), had lower mean BMI (*p* < 0.001 for both comparisons), and higher mean physical activity levels (*p* < 0.001 for both comparisons). Non-Hispanic white participants consumed more fruits and vegetables than Asian participants or participants of other races/ethnicities (*p* = 0.011 and *p* = 0.016, respectively). Asian participants consumed less fried food than non-Hispanic white or participants of other races/ethnicities (*p* < 0.001 for both comparisons). Smoking rates significantly differed by sex only for the Asian participants (6% of Asian women had ever smoked versus 61% of Asian men).

Blood pressure and hypertension values differed by race/ethnicity, statin medication use, and diabetes status (Table 1). Asian participants had a significantly higher mean SBP and PP than non-Hispanic white participants (*p* = 0.002 for both comparisons). Asians also had significantly higher mean PP than participants who self-identified with other racial/ethnic groups (*p* = 0.007). Nevertheless, both Asians and non-Hispanic whites had significantly lower mean DBP than other participants (*p* = 0.003 and *p* < 0.001, respectively). Non-Hispanic whites also had a significantly lower prevalence of hypertension compared to either Asians or other participants (*p* = 0.009 and *p* = 0.022, respectively). Participants taking statin medications had higher mean SBP, higher mean PP, and a higher hypertension prevalence than participants who were not taking statins (*p* < 0.001 for all comparisons). Similarly, participants with diabetes had higher mean SBP, higher mean PP, and a higher hypertension prevalence than participants without diabetes (*p* = 0.003, *p* < 0.001, and *p* < 0.001, respectively).

Annual average levels of TAA-PNC were between 9000 and 35,000 particles/cm^3^. The mean TAA-PNC was 22,000 particles/cm^3^ while the median TAA-PNC was 23,000 particles/cm^3^ (interquartile range = 9000 particles/cm^3^). Mean natural log-transformed TAA-PNC was significantly higher among Asian participants than among non-Hispanic white participants (*p* < 0.001; Table 1). Exposure levels were also significantly higher among participants taking statins than among those not taking statin medications (*p* = 0.022). There was no difference in mean exposure level by diabetes status (*p* = 0.579).

In our main analysis, we found positive, non-significant associations between natural log-transformed TAA-PNC exposure with SBP (β = 5.23, 95% CI = −0.68, 11.14 mmHg per natural log-unit increase), PP (β = 4.27, 95% CI = −0.79, 9.32 mmHg), and hypertension prevalence (OR = 1.81, 95% CI = 0.94, 3.48; Table 2). We found less evidence for an association with DBP (β = 0.96, 95% CI = −2.08, 4.00 mmHg). For reference, the difference in TAA-PNC concentration for a participant 0.5 natural log-units above the mean compared to a participant 0.5 natural log-units below the mean is approximately 21,000 particles/cm^3^. The results were not sensitive to the choice of the proxy for diet if fruit and vegetable consumption was used, though the associations were attenuated when race/ethnicity was used as a proxy (Table 2; Models C and D). The results were sensitive to the assumption that proximity was accounted for within the exposure value. Additional adjustment for proximity-related variables attenuated the associations, though adjusting for annoyance with traffic sound did not change the primary results (Table 2; Models F–H).

### 3.1. Effect Modification

**Figure 2:**
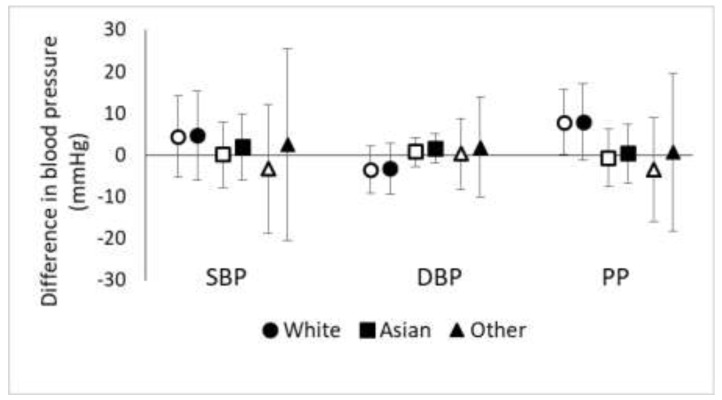
Effect modification of the association of ln(TAA-PNC) with blood pressure by race/ethnicity. Open markers represent unadjusted associations (Model A) while solid markers represent adjusted associations (Model B; adjusted for BMI, sex, smoking, physical activity, and diet as fried food consumption).

We found some evidence that race/ethnicity modified the relationship between natural log-transformed TAA-PNC and blood pressure (Figure 2). Specifically, there seemed to be stronger associations among non-Hispanic white participants than among other participants. For SBP, PP, and hypertension prevalence, these were stronger positive associations while for DBP, there was a stronger inverse association (Figure 2; OR for hypertension among non-Hispanic whites = 3.47, 95% CI = 0.83, 14.5; OR for Asians = 1.09, 95% CI = 0.42, 2.83; OR for participants of other races/ethnicities = 0.52, 95% CI = 0.03, 10.44). Adjustment for age generally made the effect estimates for non-Hispanic whites slightly stronger while making the effect estimates for other participants slightly weaker (results not shown).

**Figure 3:**
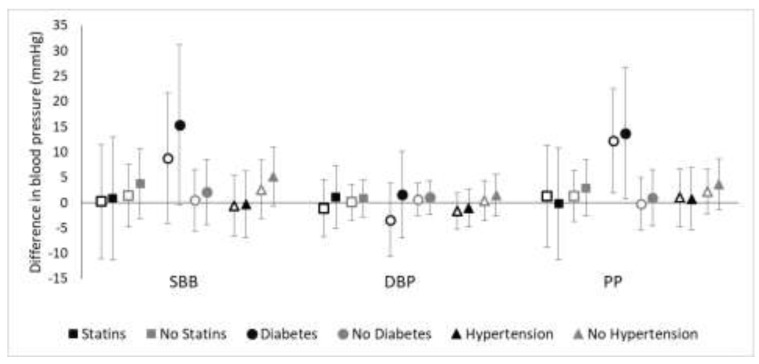
Effect modification of the association of ln(TAA-PNC) with blood pressure by statin medication use, diabetes status, and hypertension status. Open markers represent unadjusted associations (Model A) while solid markers represent adjusted associations (Model B; adjusted for BMI, sex, smoking, physical activity, and diet as fried food consumption).

We found little evidence of effect modification of the relations of natural log-transformed TAA-PNC with blood pressure by statin medication use (Figure 3). While the SBP and PP point estimates were slightly higher for participants not on statins, the confidence intervals overlapped completely. There was more evidence that having diabetes and, to a lesser extent, not having hypertension strengthened the relation of natural log-transformed TAA-PNC with SBP and PP (Figure 3). No evidence existed for effect modification of the relation of natural log-transformed TAA-PNC with DBP or hypertension for either statin medications (OR for statin users = 3.54; 95% CI = 0.55, 22.94; OR for non-users = 1.28; 95% CI = 0.61, 2.68) or diabetes status (OR for diabetics = 2.29; 95% CI = 0.31, 16.97; OR for non-diabetics = 1.63, 95% CI = 0.79, 3.38). Additional adjustment for age generally did not substantially change any of the conclusions, although it widened the already wide confidence intervals.

### 3.2. Consistency of Associations over Time

**Figure 4:**
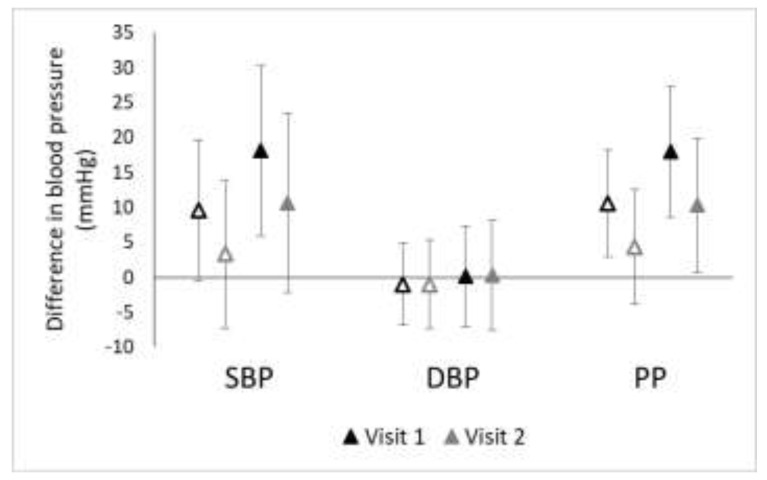
Effect estimates for the association of ln(TAA-PNC) with blood pressure measures for participants in Somerville and Dorchester/South Boston who attended two clinic visits (*n* = 205). Open markers represent unadjusted associations (Model A) while solid markers represent adjusted associations (Model B; adjusted for BMI, sex, smoking, physical activity, and diet as fried food consumption).

Of the 205 participants who attended two clinic visits approximately five months apart (all of whom resided in Somerville or Dorchester/South Boston), 67% identified as non-Hispanic white and 4% identified as Asian. Mean SBP decreased by 4.5 mmHg (95% CI = 2.4, 6.5 mmHg decrease), mean DBP decreased by 2.8 mmHg (95% CI = 1.6, 4.1 mmHg decrease), and mean PP decreased by 1.6 mmHg (95% CI = 0.2, 3.1 mmHg decrease). We found that the effect estimates for the association of natural log-transformed TAA-PNC with SBP, PP, and hypertension weakened slightly from clinic visit one to clinic visit two (Figure 4; OR for visit one = 10.3, 95% CI = 1.7, 60.9; OR for visit two = 7.0, 95% CI = 1.4, 36.4). Additional adjustment for proximity did not change these trends (results not shown).

## 4. Discussion

We found that long-term exposure to UFP (measured as TAA-PNC) was positively, though not significantly, associated with SBP, PP, and hypertension prevalence. The observed associations correspond to differences in SBP and PP that are approximately equivalent to differences observed with an increase of 3–9 years of age [35]. The associations were stronger among participants who identified as non-Hispanic white than among participants who identified as Asian or as another race/ethnicity. Additionally, the associations with SBP and PP were stronger among participants with diabetes than among participants without diabetes. They were also slightly stronger among participants without hypertension than among participants with hypertension. We did not find evidence of an association of UFP with DBP overall or among any sub-group.

As expected, our results were consistent with a previous analysis of the association between long-term UFP exposure and hypertension among adults participating in CAFEH (OR = 1.28, 95% CI = 0.81, 2.02 for the previous analysis compared to our result of OR = 1.81, 95% CI = 0.94, 3.48) [25]. The primary difference in these analyses was how hypertension prevalence was defined; in the previous analysis, elevated SBP and DBP measurements were not considered as part of the diagnostic criteria (which also modestly changed the sample size). Additionally, the covariates included in the previously published paper were not chosen based on a conceptual model, such as a DAG [25].

Our results were also generally consistent with the emerging evidence from longitudinal studies that long-term exposure to UFP is associated with cardiovascular impacts. For example, several longitudinal studies have reported positive associations with biomarkers of inflammation and other sub-clinical cardiovascular markers [14,15,16,17]. Additionally, our finding that long-term exposure to UFP was positively associated with hypertension prevalence is similar to the findings from a Canadian cohort that long-term UFP exposure is associated with incident hypertension, even adjusting for PM_2.5_ and nitrogen dioxide exposure [18].

Nevertheless, our finding in this cross-sectional study that long-term UFP exposure was positively, but not significantly, associated with SBP and PP was not consistent with our previous finding in a prospective study of Puerto Rican adults residing in eastern Massachusetts. In that study, we found that long-term exposure to UFP was not associated with changes in SBP (β = 0.96; 95% CI = − 0.33, 2.25 mmHg) or PP (β = 0.70; 95% CI = − 0.27, 1.67 mmHg) [16]. Our present study is cross-sectional, and exposures were assessed in the year of the clinical examination (rather than exclusively before blood pressure measurements were taken) and as such, the present study cannot address the question of whether UFP exposure could be considered a causal risk factor for changes in blood pressure. The idea that there could be exposure misclassification or a temporal misalignment in the present study was supported in that the effect estimates were attenuated among the sub-set of participants who attended a second clinic visit approximately five months after their primary clinic visit (Figure 4). Some of the instability of the effect estimates could be due to seasonal differences as particle composition can vary with time of year [36,37] and blood pressure tends to be higher when temperatures are cooler (77% of the first clinic visits occurred between October and March while 96% of the second visits occurred between April and September) [38,39]. It is also possible, however, that some of the differences between the previous study and this one reflect differences in the study populations. In the present analysis, only 6% of participants were Hispanic while in the previous analysis, all participants identified as of Puerto Rican descent.

In the present study, we found evidence that the associations with UFP differed among sub-groups. For example, we found somewhat stronger associations of UFP with SBP, PP, and hypertension among non-Hispanic whites than among other participants, despite higher UFP exposures and increased exposure contrast among participants who identified as Asian or as another race/ethnicity. Given the strong spatial segregation of participants in our study areas by race/ethnicity, it is possible that the differences we observed were attributable to other neighborhood social or environmental characteristics rather than to UFP [40,41,42]. This idea was partially supported in that the associations were attenuated when we controlled for race/ethnicity, which in the CAFEH population is strongly associated with both exposure to social and environmental factors and to differences in health status [26]. It is also possible that differences in general health status modified the associations of UFP with the health outcomes. In previous studies, as in our study, co-morbidities and medication use affected the strength of associations [16,17]. It was a strength of our present study that we were able to recruit a diverse population from several communities in Boston. This allowed us to consider the relationship of UFP with BP outcomes among sub-sets of the population defined by race/ethnicity and by health status.

Another strength of our study was our use of a conceptual model to identify a minimally sufficient adjustment set of covariates. We were able to explicitly state and test our assumptions about the factors that confound the relationship between UFP and BP. For example, if we had included a direct path from sex to cooking (rather than only an indirect path), the minimally sufficient adjustment set of covariates would not change. Similarly, if we included a direct path from age to education to reflect changes in educational attainment patterns over time, the minimally sufficient adjustment set would not change. Nevertheless, if we had included a direct path from age to cooking, age should be included in the minimally sufficient set. As Model E results show, this would slightly attenuate the associations of UFP with SBP and PP.

The use of the conceptual model also allowed us to test the sensitivity of our analyses to the choice of proxy for variables represented within the minimally sufficient adjustment set. For example, we compared the results using fried food consumption and fruit and vegetable consumption as proxies for diet and found that the results were robust against choice of dietary variable. This was important because these two dietary components represented different dietary patterns within our study population (e.g., non-Hispanic whites consumed more fried food and fruits and vegetables, Asians consumed fewer fried foods and fruits and vegetables, and participants of other races/ethnicities consumed more fried foods but fewer fruits and vegetables). Similarly, we were able to quantitatively assess our assumption that since UFP exposure was assessed as a function of distance to roadways, additional adjustment for proximity would result in over-adjusted models. We found that adjustment for annoyance from traffic sound did not meaningfully change the results and that adjustment for other proxies for proximity resulted in attenuated associations, as would be expected if the models were over-adjusted.

Our exposure assessment strategy had both strengths and limitations. Our UFP model was finely resolved in space (20-m resolution) and time (hourly resolution). We further adjusted our exposure estimates for time spent in different micro-environments, avoiding the bias introduced by assuming residential exposure concentrations are representative of long-term UFP exposure [20]. While we did not control for short-term UFP exposures and we did not account for short-term changes in blood pressure, previous work has suggested that controlling for short-term UFP exposure is unlikely to meaningfully change the results [15] and our group has found that daily average UFP exposure is not associated with SBP or PP (though it is associated with DBP) in the CAFEH population [43]. There could still be concerns about the exposure window we used. While UFP exposures were assigned for the calendar year of the study visit, a more relevant time window would include only time preceding the study visit. Additionally, to the extent that exposure misclassification varied by neighborhood or by time period, our analyses of effect modification by race/ethnicity would be strongly impacted due to the spatial segregation of participants and the fact that we collected data from different neighborhoods in different years. Finally, although other air pollutants could act independently of or jointly with UFP to affect blood pressure, we were unable to adjust for exposure to other air pollutants. Beyond the exposure assessment limitations, the major limitations of our study include the cross-sectional design, the fairly small sample size, and the multiple comparisons we made increasing the likelihood of a Type I error. The associations we observed with UFP should not be interpreted causally unless future work addressing the limitations confirms our findings.

## 5. Conclusions

Overall, we found evidence that UFP was positively associated with SBP, PP, and hypertension prevalence. While our study adds to the growing body of literature on the association between long-term exposure to UFP and cardiovascular outcomes, future longitudinal research should consider the impact of long-term UFP exposure on incident hypertension and changes in BP over time in diverse populations. 

## Figures and Tables

**Table 1:** Study sample characteristics.

	Total		White		Asian		Other	
Characteristic	*n*	*% (n)* *or mean (sd)*	*n*	*% (n)* *or mean (sd)*	*N*	*% (n)* or *mean (sd)*	*N*	*% (n)* or *mean (sd)*
TAA-PNC * (particles/cm^3^)	409	22,000 (6500)	178	20,000 (4900)	149	24,000 (7900)	82	21,000 (5000)
ln[(TAA-PNC) (particles/cm^3^)]	409	9.9 (0.35)	178	9.8 (0.28)	149	10.0 (0.43)	82	9.9 (0.27)
SBP (mmHg)	409	137.5 (19.5)	178	133.9 (18.3)	149	141.2 (20.6)	82	138.9 (18.8)
DBP (mmHg)	409	77.7 (10.3)	178	76.2 (10.7)	149	77.3 (9.3)	82	81.9 (10.4)
PP (mmHg)	409	59.8 (16.5)	178	57.6 (14.9)	149	63.9 (18.4)	82	57.1 (15.1)
Hypertension	409	63.8 (261)	178	55.6 (99)	149	69.8 (104)	82	70.7 (58)
Age (years)	409	61.5 (12.8)	178	59.8 (11.3)	149	66.6 (13.4)	82	56.0 (11.3)
BMI (kg/m^2^)	393	27.7 (6.8)	168	29.5 (6.9)	149	24.1 (4.1)	76	30.6 (7.7)
ln[light/moderate physical activity (min/week)]	374	4.3 (2.2)	164	3.8 (2.3)	147	5.1 (1.6)	63	3.5 (2.3)
Female	409	59.2 (242)	178	59.6 (106)	149	56.4 (84)	82	63.4 (52)
Smoker status	398		176		145		77	
*Current*		21.1 (84)		22.2 (39)		14.5 (21)		31.2 (24)
*Former*		30.7 (122)		43.2 (76)		15.9 (23)		29.9 (23)
*Never*		48.2 (192)		34.7 (61)		69.7 (101)		39.0 (30)
Fruit and vegetable consumption ≥ 7x/week	275	38.2 (105)	125	48.0 (60)	97	30.9 (30)	53	28.3 (15)
Fried food consumption ≥ 1x/week	405	33.8 (137)	176	45.5 (80)	149	14.8 (22)	80	43.8 (35)
Educational Attainment	409		178		149		82	
*< HS*		34.2 (140)		11.2 (20)		61.7 (92)		34.2 (28)
*HS*		31.8 (130)		36.5 (65)		24.2 (36)		35.4 (29)
*>HS*		34.0 (139)		52.3 (93)		14.1 (21)		30.5 (25)
Born in the USA	404	45.5 (184)	174	86.8 (151)	149	0.0 (0)	81	40.7 (33)
Statin Medications	400	29.0 (116)	176	31.3 (55)	144	29.2 (42)	80	23.8 (19)
Hypertension medications	400	45.0 (180)	176	35.8 (63)	144	54.2 (78)	80	48.8 (39)
Diabetes	399	20.3 (81)	175	17.7 (31)	144	18.8 (27)	80	28.8 (23)

* Time-activity-adjusted particle number concentration. Italics indicate variable levels.

**Table 2:** Effect estimates for ln (TAA-PNC).

Model	SBP (mmHg)	DBP (mmHg)	PP (mmHg)	Hypertension
	**β (95% CI)**	**β (95% CI)**	**β (95% CI)**	**OR (95% CI)**
Model A	2.87 (−2.60, 8.33)	−0.18 (−3.09, 2.72)	3.05 (−1.58, 7.68)	1.53 (0.86, 2.72)
**Model B**	**5.23 (**−**0.68, 11.14)**	**0.96 (**−**2.08, 4.00)**	**4.27 (**−**0.79, 9.32)**	**1.81 (0.94, 3.48)**
Model C	5.84 (−1.94, 13.61)	1.79 (−1.95, 5.52)	4.05 (−2.80, 10.90)	1.53 (0.68, 3.43)
Model D	2.41 (−3.51, 8.32)	0.15 (−2.92, 3.21)	2.26 (−2.83, 7.35)	1.27 (0.64, 2.52)
Model E	3.60 (−1.75, 8.95)	1.10 (−1.95, 4.15)	2.50 (−1.74, 6.74)	1.72 (0.84, 3.55)
Model F	1.67 (−3.87, 7.22)	0.01 (−3.15, 3.18)	1.66 (−2.78, 6.11)	1.25 (0.57, 2.75)
Model G	1.68 (−3.89, 7.24)	−0.19 (−3.35, 2.97)	1.86 (−2.58, 6.31)	1.31 (0.59, 2.91)
Model H	5.67 (−0.40, 11.75)	1.35 (−1.76, 4.45)	4.33 (−0.88, 9.53)	1.86 (0.95, 3.66)

(A) Unadjusted (*n* = 409). (B) Main model adjusted for BMI, sex, smoking, physical activity, and diet (as fried food consumption; *n* = 347). (C) Model B covariates but using fruit and vegetable consumption for diet (*n* = 237). (D) Model B covariates but using race/ethnicity for diet (*n* = 350). (E) Model B covariates as well as age (*n* = 347). (F) Model B covariates as well as additional adjustment for proximity (as race/ethnicity, age, educational attainment; *n* = 347). (G) Model B covariates as well as additional adjustment for proximity (as race/ethnicity, age, educational attainment, random or convenience sample participant; *n* = 347). (H) Model B covariates as well as additional adjustment for proximity (as annoyance at traffic sound; *n* = 344). Model B is bolded since it is the main model.

## Appendix A: Exposure Assessment

In each of Somerville, Dorchester/South Boston, Chinatown, and Malden, we drove the Tufts Air Pollution Monitoring Laboratory (TAPL-1) around a fixed route 2–6 times/day on 35 days in the same year in which we collected health information from participants. Monitoring occurred in all four seasons, on all days of the week, and at different times of the day (04:00–22:00). Data quality assurance and control steps have been described previously [23,31,44]. Community-specific multivariate regression models predicting log-transformed hourly PNC were developed using spatial (e.g., side of and distance to highway) and temporal (e.g., wind speed and direction) covariates. Model performance was adequate (model adjusted-R^2^: Somerville = 0.42, Dorchester/South Boston = 0.35, Chinatown = 0.23, and Malden = 0.31) and stable under leave-one-day-out cross-validation [23].

Using the community-specific PNC models, hourly residential ambient PNC were estimated for each participant for every hour of the year [24]. These estimates were then adjusted for the amount of time individual participants reported spending on a workday/weekday and a non-workday/weekend day in five micro-environments (inside home, outside home, at work, on highway, and other). For hours spent inside the home when local meteorological stations reported ambient temperatures exceeding 21 °C (70 °F), the hourly residential value was modified to account for air conditioner use (window or central air conditioners, depending on survey data). For hours spent at work in occupations that included traffic-related air pollution exposure (e.g., bus drivers), the hourly residential ambient PNC value was replaced with the mean hourly residential PNC of all participants residing ≤ 50 m from the highway. For hours spent at work in occupations without substantial traffic-related air pollution exposure (e.g., nurses) and for time spent in “other” micro-environments, the hourly residential ambient PNC value was replaced with the mean hourly residential PNC of all participants residing > 1000 m from the highway. For time spent on the highway, we replaced the hourly residential PNC with the on-highway concentration predicted by the Somerville model. Individual time-activity-adjusted (TAA) PNC values for each hour were averaged to assign annual TAA-PNC for each participant [20,24].

## Appendix B: Directed Acyclic Graph Justification

To develop the directed acyclic graph (DAG), we first identified consensus statements [2,45,46,47,48], meta-analyses [49,50,51], systematic reviews [52,53], reviews [54,55,56,57,58,59,60,61], and longitudinal cohort studies [62,63,64,65,66,67] assessing determinants of blood pressure in adults. We also conducted a PubMed search in January 2017: ((((“blood pressure”[Title]) OR “hypertension”[Title]) AND ((“meta analysis”[Publication Type] OR “review”[Publication Type]))) AND (“risk factor”[Title] OR “risk factors”[Title] OR “determinant”[Title] OR “determinants”[Title] OR “effect of”[Title] OR “influence of”[Title] OR “impact of”[Title])) AND english[Language] Filters activated: Humans, Adult: 19+ years. This search resulted in 94 titles. From a review of the abstracts in Abstrackr [68], we kept 30 articles [69,70,71,72,73,74,75,76,77,78,79,80,81,82,83,84,85,86,87,88,89,90,91,92,93,94,95,96,97]. Articles were excluded if they focused primarily on early life or genetic risk factors, blood pressure or hypertension as an exposure, hypertension treatment or management, blood pressure in children or adolescents, pregnancy-related hypertension, or treatment-resistant hypertension.

From these articles, we identified 21 risk factors for hypertension and high blood pressure (acculturation/migration, age, alcohol, BMI, caffeine/coffee/cocoa, DASH diet, diabetes, dietary fiber/protein supplementation, education/SES, estradiol, glucocorticoids, physical activity, potassium, race, racial discrimination, sex, smoking, sodium intake, statins, stress, and vitamin C). We examined the pairwise relationships between UFP and each of the identified risk factors [19,98,99,100,101,102,103,104,105,106,107]. We prioritized evidence from meta-analyses, systematic reviews, reviews, or consensus statements that addressed direct and indirect (within two steps) determinants of personal exposure to UFP. If these types of articles were not available, we considered evidence from experimental studies, cohort studies, and other study designs (in that order). We used the same evidence prioritization method to assess the relationships among potential covariates [90,108,109,110,111,112,113,114,115,116,117,118,119,120,121,122,123,124,125,126,127,128,129,130,131]. We also considered relationships that were introduced by the study design. For example, convenience sampling was done in specific retirement communities so age was considered an ancestor of UFP exposure. Based on all of the pairwise relationships, we built the DAG in DAGitty v2.3 [132]. The code for the DAG is below.

Of the 99 testable conditional independencies suggested by the DAG, 69 held within our dataset when using the variable for fried food consumption as a proxy for diet and 70 held when using the variable for fruit and vegetable consumption. The most problematic covariate was the proxy for cooking (gas stove use) which is due partially to the fact that none of the Somerville participants responded to this question. Statements of conditional independence with UFP did not hold in our dataset for race, education, and convenience sampling (each with three different sets of covariates) or for acculturation/migration, age, and fruit/vegetable intake (each with one set of covariates).

**DAG Code:**

Acculturation%2Fmigration 1 @−0.848, −2.923

Age 1 @−4.724, −2.738

BMI 1 @1.013, −1.037

Blood%20pressure O @3.740, 5.031

Convenience%20sample 1 @−4.594, 1.461

Cooking 1 @−2.922, 3.995

Diet 1 @−1.535, 3.699

Education%2FSES 1 @0.966, −2.664

Inhalation%20rate 1 @−3.610, 3.033

Physical%20activity 1 @−1.998, 1.109

Proximity 1 @−4.866, 3.273

Race 1 @−3.302, −1.888

Sex 1 @2.068, −0.630

Smoking 1 @−3.586, 0.813

UFP E @−4.700, 4.920

Acculturation%2Fmigration BMI Blood%20pressure Diet Physical%20activity Proximity Smoking

Age Acculturation%2Fmigration BMI Blood%20pressure Convenience%20sample Diet Physical%20activity Smoking

BMI Blood%20pressure Inhalation%20rate

Convenience%20sample Proximity

Cooking UFP

Diet BMI Blood%20pressure Cooking

Education%2FSES BMI Blood%20pressure Diet Physical%20activity Proximity Smoking

Inhalation%20rate UFP

Physical%20activity BMI Blood%20pressure Inhalation%20rate

Proximity UFP

Race Acculturation%2Fmigration BMI Blood%20pressure Convenience%20sample Diet Education%2FSES Physical%20activity Proximity Smoking

Sex BMI Blood%20pressure Diet Inhalation%20rate Physical%20activity Smoking

Smoking Blood%20pressure Inhalation%20rate UFP

UFP Blood%20pressure
